# From Friend to Foe: Toxicity Trade-Offs Govern *Staphylococcus aureus* Infection Severity

**DOI:** 10.1371/journal.pbio.1002230

**Published:** 2015-09-02

**Authors:** Lauren A. Richardson

**Affiliations:** Public Library of Science, San Francisco, California, United States of America

## Abstract

The life-threatening human pathogen *Staphylococcus aureus* experiences an evolutionary tug-of-war between highly toxic strains, which are better able to transmit between hosts and less toxic strains which are better at infecting a single host. Read the Research Article.

*Staphylococcus aureus* is a nearly ubiquitous bacterium. Most of the time, it lives quietly and unnoticed as a member of the microbiota on your skin and in your nose. However, *S*. *aureus*—or “staph” as it is commonly known—is an opportunistic pathogen, switching from friend to foe when it gets the chance. Some staph infections can be mild, local infections while in other instances it can lead to severe, life-threatening cases of pneumonia and bacteremia (blood infection) (Fig 1).

**Fig 1:**
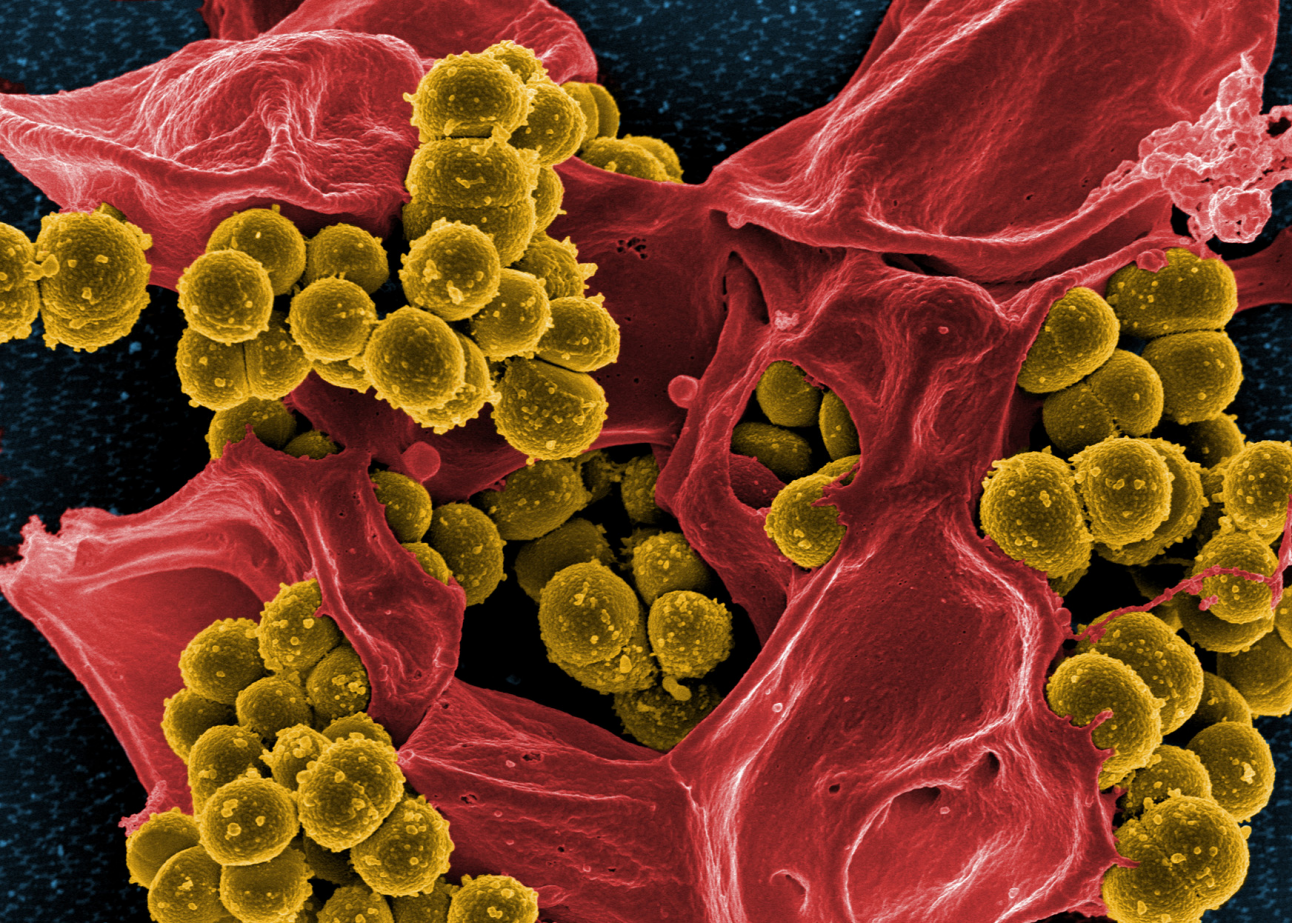
Methicillin-resistant *S*. *aureus* (MRSA). *S*. *aureus* is a ubiquitous commensal bacterium and an opportunistic pathogen. When it acquires antibiotic resistance genes, it is extremely difficult to treat. *Image credit*: *NIAID*.

When staph goes from being a mild-mannered microbiota member (a state known as carriage) to an active infection, what governs whether it will be a minor or a major infection? Previous research has implicated the secretion of toxins by staph as one of the determinants. These toxins are responsible for damaging local tissue, releasing nutrients to aid in bacteria growth, and blocking the host immune response. Toxins are also critical to the transmission of staph both within and between hosts. Thus, it seems to make inherent sense that strains of staph that secrete lots of toxins might be more likely to cause severe infections, and most research to date, particularly in mouse models, has supported this idea.

However, the idea that high toxin secretion causes severe infection is now in doubt, based on a study published in *PLOS Biology* by Maisem Laabei, Daniel Wilson, Mario Recker, Ruth Massey, and coworkers. Their work, using functional genomics and mathematical modeling, suggests that it is the low toxin–expressing strains that are most likely to establish severe infections.

In their paper “Evolutionary Trade-Offs Underlie the Multi-Faceted Virulence of *Staphylococcus aureus*,” the authors use two collections of clinical staph samples to investigate the link between toxicity and virulence. The first collection is a series of samples taken from a single patient as they progressed from carriage to a severe bacteremia infection over the course of several months. When the authors looked at the toxicity of these samples, they found that during the carriage phase the staph strains secreted more toxins than during the bacteremia phase. The second collection were all isolates of the same strain of staph (the infamous USA300 strain) taken from multiple patients with varying degrees of infection. Again, the authors found that carriage and mild infections were far more likely to be caused by high toxin producing strains and that severe infections were more often established by low toxin producing strains.

These counterintuitive findings beg the question of why low toxin strains might be better able to establish severe infections. While the underlying health of the patient certainly contributes to the susceptibility of developing a severe infection, it does not explain why patients are more likely to succumb to bacteremia with the less toxic strains of staph. The authors were also able to rule out several of the more obvious theories, such as that low toxin–producing strains are better at invading cells or eluding the immune system than high toxin–producing strains.

The only difference that the authors were able to find between the low- and high-toxin strains was their ability to grow in human serum. The bloodstream is a very hard place for bacteria to grow, with the entire immune system present and actively policing this niche. Additionally, serum contains many antimicrobial peptides and can neutralize staph-produced toxins. Since producing toxins is known to be energetically demanding, and serum induces toxin production, the authors hypothesized that the cost of toxin production might disadvantage these strains in this challenging environment. To test this, they grew high- and low-toxin strains in a rich broth with and without added human serum. They saw that in the absence of serum, strains had equivalent fitness, but when serum was added, the low-toxin strains were able to outperform the high-toxin strains. This suggests that low toxin–producing strains are able to establish blood infections because they do not waste energy producing toxins and thus have more energy for defense and growth.

But while these findings suggest an answer to why the low-toxin strains are better able to establish a severe infection, they raise another question: why do high toxin–producing strains exist at all? If low-toxin strains are fitter than high-toxin strains, then evolutionary pressure should have purged all high-toxin strains long ago. To understand this conundrum, the authors created a simple mathematical model. Their model accounts for the observations that highly toxic strains have increased transmissibility and are better at progressing from carriage to a mild infection. On the other hand, while low toxin strains are better at establishing bacteremia, they have much worse transmissibility, i.e., a blood infection is very rarely passed on to another person. Their modeling suggests that the lower transmission rate of less toxic strains could contribute to the maintenance of highly toxic strains at the population level.

The results of this study paint a complex picture of staph virulence. There appears to be a genetic tug of war between highly toxic strains, which are better able to transmit between hosts, and less toxic strains, which are better at infecting a single host. Add to this that a severe infection by a low-toxin strain is a transmission dead-end, and that this (theoretically) leads to an evolutionary selection for higher levels of toxicity—and thus transmissibility—in the population. This trade-off between virulence and toxin expression can at least partially explain why staph can be both a constant constituent of the microbiota and a deadly pathogen.
